# Vacuum-Assisted Breast Biopsy and Post-Biopsy Scarring: Implications for Imaging Interpretation

**DOI:** 10.3390/diagnostics16172705

**Published:** 2026-08-25

**Authors:** Omar El Sardouk, Joe Rizkallah, Farah Abou Zeid, Ghina Berjawi, Lara Nassar

**Affiliations:** Department of Diagnostic Radiology, American University of Beirut, Beirut P.O. Box 11-0236, Lebanon; oe34@aub.edu.lb (O.E.S.); jr56@aub.edu.lb (J.R.); fa139@aub.edu.lb (F.A.Z.); gb02@aub.edu.lb (G.B.)

**Keywords:** vacuum-assisted breast biopsy, breast cancer, mammography, scar formation, tomosynthesis, biopsy markers

## Abstract

**Background/Objectives**: Breast cancer is the most common malignancy among women worldwide. Vacuum-assisted breast biopsy (VABB) has emerged as a minimally invasive alternative to surgical excision, but it may cause post-biopsy scarring that could mimic or obscure malignancies on follow-up imaging. This study aimed to evaluate the imaging characteristics, evolution, and clinical implications of post-VABB scar formation. **Methods**: We conducted a retrospective study of patients who underwent mammographic or magnetic resonance imaging-guided VABB at our institution between January 2010 and December 2020 and whose follow-up report describes a post-biopsy scar. Patients with malignant or high-risk lesions requiring subsequent surgery or prior breast surgery were excluded. Post-biopsy follow-up mammograms and ultrasounds were reviewed by a fellowship-trained breast radiologist for scar formation, visibility across imaging modalities, and evolution over time. Statistical analysis was performed to assess associations between scar detection and patient or lesion characteristics. **Results**: Thirty-seven patients (39 lesions) with documented post-biopsy scarring on follow-up imaging reports met the study inclusion criteria. The mean age at biopsy was 51.97 ± 12.40 years. Lesions were almost equally distributed between the right and left breasts, with 19 cases involving the right breast (48.7%) and 20 cases involving the left breast (51.3%). Most cases did not need any workup, except for six cases, where two required further biopsy and four required short-term follow-up. No malignancy was detected during the available follow-up or additional evaluation. Twenty-two scars regressed over time. The estimated mean time to scar regression was 69.47 months. **Conclusions**: Post-biopsy scarring was documented in a small subset of our institution’s VABB cases over the study period and may resemble malignant findings, particularly on tomosynthesis. Awareness of typical scar characteristics, careful documentation of biopsy sites, and correlation with clip markers are essential to avoid unnecessary interventions.

## 1. Introduction

**Figure 1:**
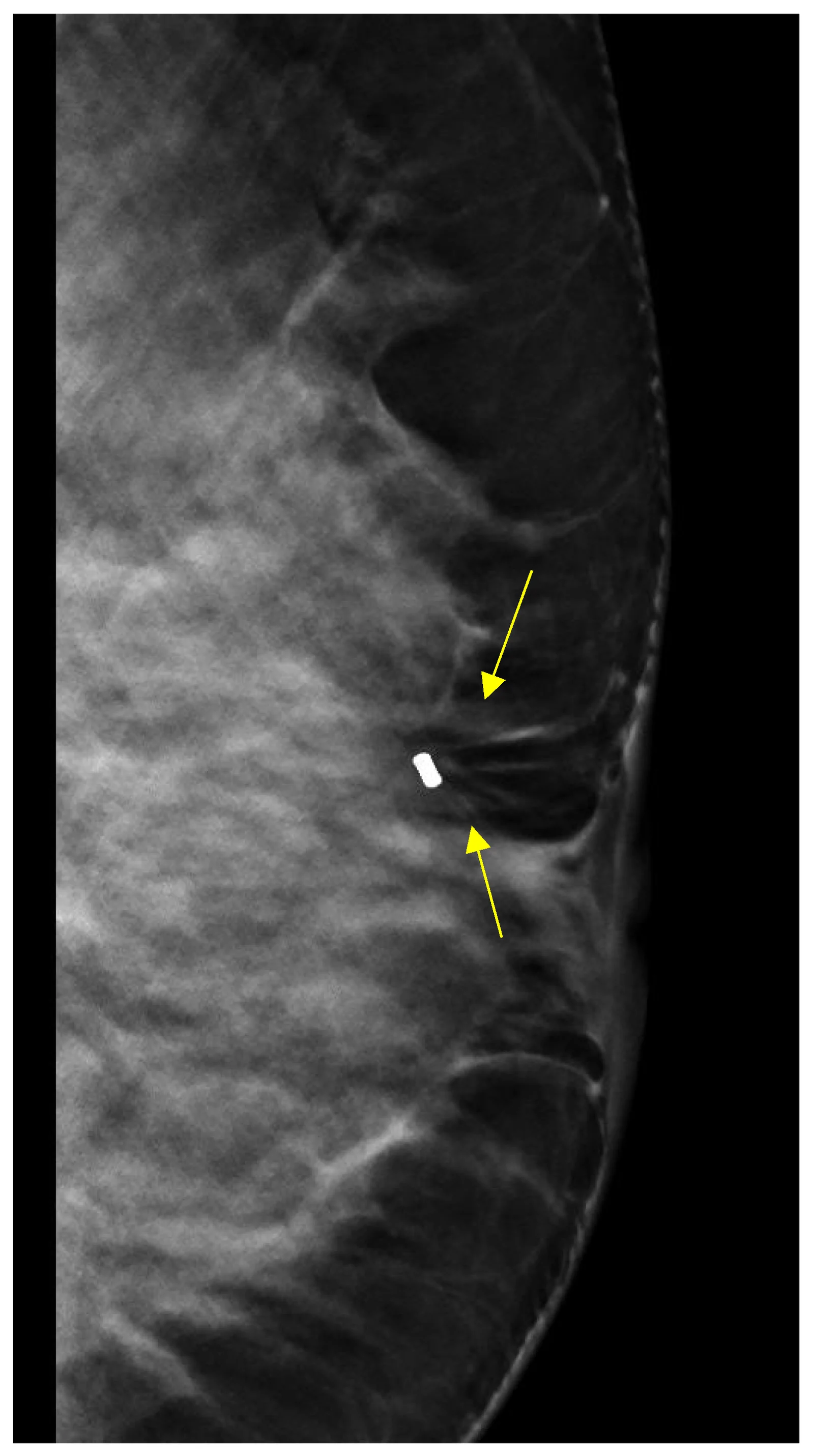
Slice from a tomosynthesis left MLO view in a patient status post-benign biopsy with a clip marker in place. The available view demonstrates mild distortion (arrows) adjacent to the clip marker, indicating post-biopsy scarring.

Breast cancer has the highest incidence and mortality in females among all cancers globally [1]. In Lebanon, 2161 new cases of breast cancer were diagnosed in 2020 alone, with the median age being 52–52.5 years [2,3]. Since the 1970s, mammograms have been developed and improved to further aid in screening for breast cancer, hence reducing mortality by up to 40% [4]. Once a lesion is suspected on mammography, a biopsy must follow. Percutaneous sampling, specifically vacuum-assisted breast biopsy (VABB), has emerged as a powerful tool for evaluating breast lesions [5,6,7,8,9]. VABB is a rotating cutter powered with suction that obtains multiple samples from the same lesion. It addresses the limitations of core biopsy and fine needle aspiration, namely the need for larger volumes of tissue, and enables biopsies of difficult-to-sample lesions [5]. Scar formation may be observed on mammograms after 2–14% of VABB; the presence of such scars must be taken into account when reading subsequent mammograms, as they may be mistaken for malignant findings or mask them (Figure 1) [5,6,10,11,12]. The literature on this subject is lacking, particularly regarding the natural history of scars and patient- or lesion-related associated factors that may affect scar formation. This study aimed to evaluate the imaging appearance, visibility, evolution over time, and clinical implications of documented scarring on follow-up imaging after VABB.”

## 2. Materials and Methods

This retrospective study was conducted on patients who underwent mammographic or magnetic resonance imaging (MRI)-guided vacuum-assisted breast biopsies between 1 January 2010 and 30 December 2020. Patients whose pathology showed malignant or high-risk lesions and in whom surgery was subsequently performed were excluded. We also excluded patients who had undergone breast surgery prior to biopsy, where post-surgical changes might have hindered the assessment of post-biopsy scarring. Only cases in which the biopsy was performed at our institution and at least one official follow-up imaging report documented post-biopsy scarring were included. One fellowship-trained breast radiologist with 14 years of experience in breast imaging reviewed all follow-up studies to assess for biopsy-related scar formation and its characteristics. The scar was defined as a new architectural distortion identified adjacent to the clip marker, or at the initial biopsy site in case the clip marker was displaced.

Vacuum-assisted breast biopsies were performed using the Suros^®^ ATEC^®^ Vacuum-Assisted Breast Biopsy System (Hologic Inc., Marlborough, MA, USA), which was originally introduced in 2003. Digital mammography examinations were performed using two full-field digital mammography systems: the Senographe Pristina (GE HealthCare, Buc, France; model in-roduced in 2016) and the Selenia Dimensions system (Hologic Inc., Marlborough, MA, USA; model introduced in 2008). The GE Senographe Pristina system was operated using software version 8.3.4 (NUEVO_1_0), while the Hologic Selenia Dimensions system used AWS software version 1.9.1.8 (DCF version 3.3.12c). Ultrasound was performed using a 5.5- to 18-MHz 18L6 HD linear transducer from the Siemens ACUSON 52000 ultrasound system (Siemens, Malvern, PA, USA).

All patients underwent biopsy using a 9G needle size. Patient age, lesion laterality, breast density, and original lesion size were recorded. Pre-biopsy lesion characteristics were recorded; those biopsied under mammographic guidance were categorized into calcifications, asymmetry, mass, and architectural distortion, and those biopsied under MRI guidance were divided into focus, mass, and non-mass enhancement. Post-biopsy follow-up mammograms and breast ultrasound examinations were reviewed. The visibility of the scar was determined by the available imaging, including 2D or 3D craniocaudal (CC) or mediolateral oblique (MLO) views and ultrasound when available.

Scar location was recorded as either corresponding to the location of the clip marker or the initial location of the biopsy site in case the clip marker was displaced. The time interval between biopsy and scar detection on follow-up imaging was measured in months. Scar visibility at the initial follow-up and its status on subsequent follow-up imaging were also recorded, with interval changes determined by visual assessment and classified as regressed, stable, or progressed. In cases where the scar prompted a workup, we documented the reason for this and whether a short-term follow-up or re-biopsy was required to evaluate the scar.

Data was analyzed to identify correlations between imaging findings, patient characteristics, and scar detection or evolution. Descriptive statistics were used to summarize patient demographics and lesion characteristics. Comparisons between categorical variables were performed using Pearson’s chi-square test when expected cell counts satisfied test assumptions and Fisher’s exact test for analyses with small expected cell counts. Kolmogorov–Smirnov and Shapiro–Wilk tests were used to test normality. Age was found to have a normal distribution; therefore, a *t*-test was used. Meanwhile, lesion size was not found to be normal, and a Mann–Whitney test was used instead. Kaplan–Meier analysis was used to estimate time for scar regression. A *p*-value < 0.05 was considered statistically significant. Statistical analyses were performed using SPSS (version 25; IBM Corp., Armonk, NY, USA).

The study was approved by the Institutional Review Board of the American University of Beirut Medical Center (protocol ID: BIO-2021-0051) and conducted in accordance with the Declaration of Helsinki. Given the retrospective nature of the medical chart review and analysis of anonymized data, the requirement for informed consent was waived by the Institutional Review Board.

## 3. Results

Between the years of 2010 and 2020, 1678 vacuum-assisted biopsies were performed in total. Their reports were reviewed, and we identified 37 patients (39 lesions) who met inclusion criteria and had post-biopsy scarring documented on follow-up imaging reports. The mean age at biopsy was 51.97 ± 12.40 years, with a median age of 51.0 years [IQR: 41.5–61.0] and an age range of 33 to 80 years. Lesions were almost equally distributed between the right and left breasts, with 19 cases involving the right breast (48.7%) and 20 cases involving the left breast (51.3%).

Sixteen cases (41%) were mammography-guided biopsies, while 23 were MR-guided biopsies, and all lesions were classified as BI-RADS 4. The mean lesion size was 16.03 ± 18.14 mm, with a median size of 9.5 mm [IQR: 6.0–19.3] and a range from 2 to 77 mm.

The breasts were heterogeneously dense in 24 cases (61.5%), extremely dense in six cases (15.3%), showed scattered fibroglandular densities in eight cases (20.5%), and were predominantly fatty in one case (2.6%).

Pre-biopsy mammographic findings most commonly consisted of calcifications, observed in 14 cases (35.9%). Less frequent findings included architectural distortion in one case (2.6%) and asymmetry in one case (2.6%). MRI findings included non-mass enhancement seen in 13 cases (33.3%), mass enhancement in seven cases (17.9%), and foci in three cases (7.7%).

All 39 biopsies were performed using a 9G needle. The biopsy clip marker was located at the biopsy site in 38 cases (97.4%), while clip displacement was reported in one case (2.6%). All included cases had benign pathology (Table 1).

The mean interval between biopsy and follow-up imaging was 18.92 ± 12.88 months, with a median follow-up delay of 14.5 months [IQR: 10.0–26.3] and a range of 2 to 49 months.

**Figure 2:**
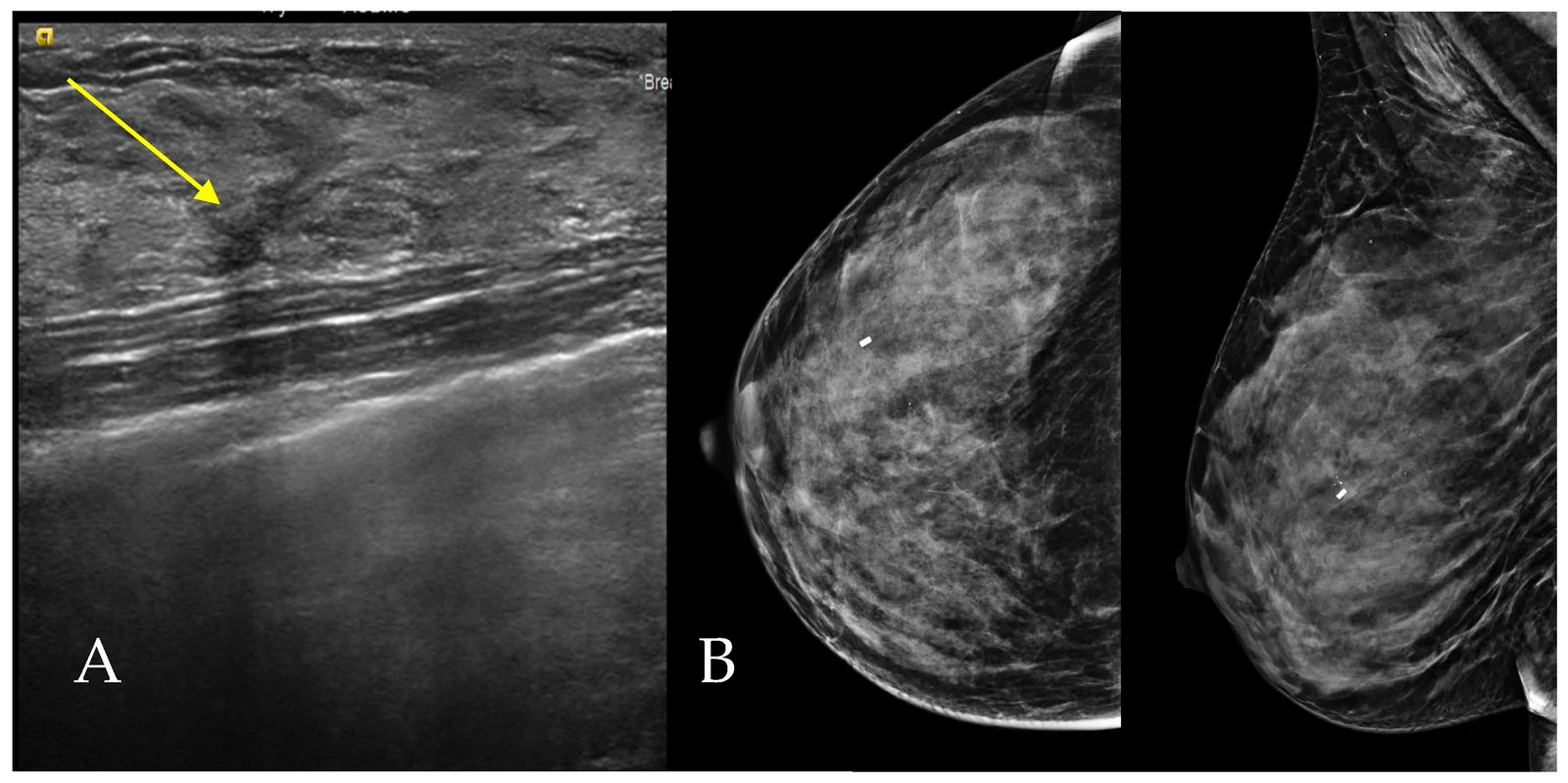
(**A**) Ultrasound of the right breast showing post-biopsy scarring appearing as a linear hypoechoic non-mass lesion adjacent to the clip marker (arrow). (**B**) 2D CC and MLO mammography of the same patient during the same visit demonstrating the clip marker but with no mammographically visible post-biopsy scarring.

**Figure 3:**
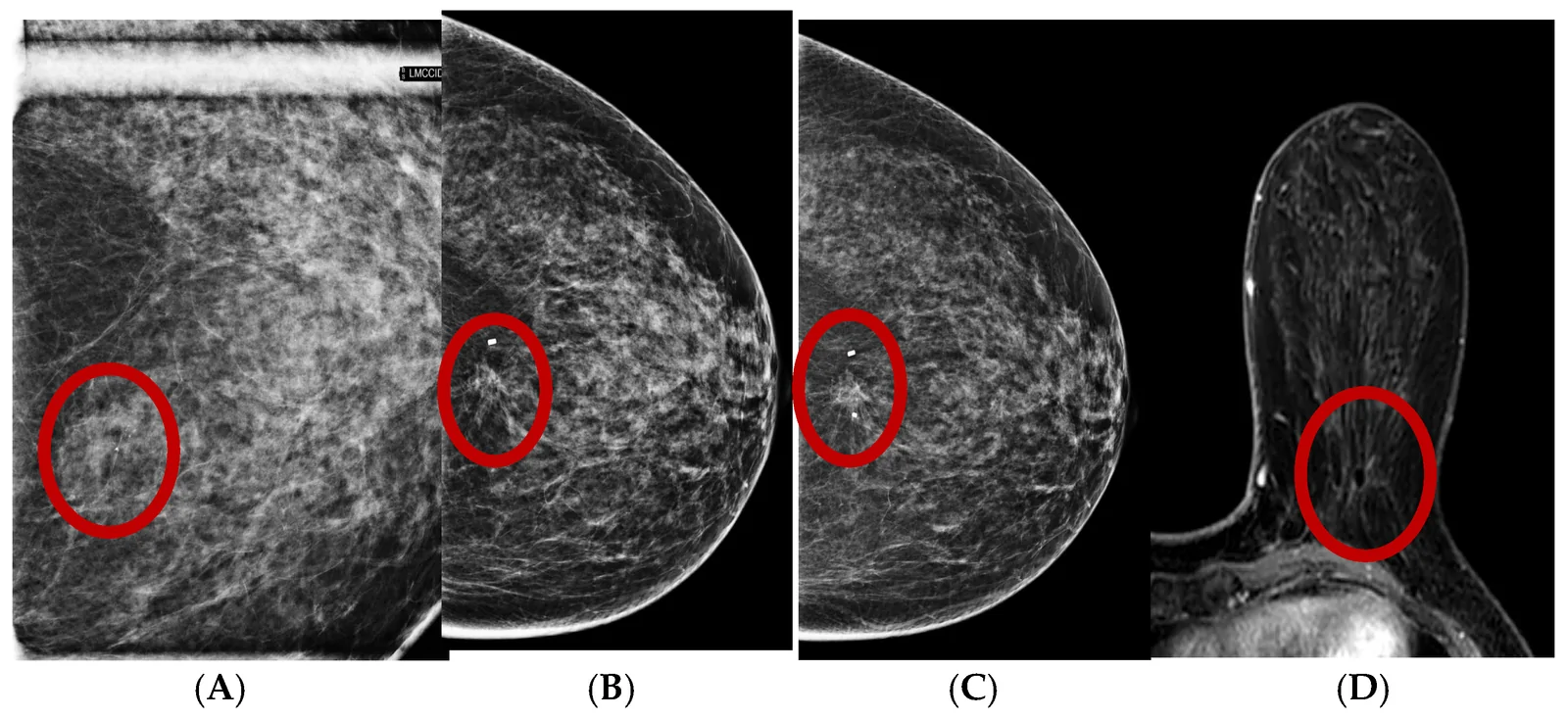
Serial breast imaging following biopsy. (**A**) Initial mammogram showing a suspicious group of microcalcifications (red circle), for which biopsy was recommended. (**B**) First post-biopsy mammogram demonstrating architectural distortion at the biopsy site, favored to represent post-procedural scarring but recommended for repeat biopsy as it was more prominent than expected for a simple post-biopsy scar. (**C**) Mammogram after the second biopsy revealing progression of the scar, identified as an increase in density at the scar site. (**D**) Breast MRI showing no abnormal enhancement, confirming the finding as benign post-biopsy scarring.

Scars were most commonly visible on 3D MLO views, reported in 32 cases (82.1%), followed by 2D CC views in 18 cases (46.2%), ultrasound in 17 cases (43.6%) (Figure 2), 2D MLO views in 14 cases (35.9%), and 3D CC views in eight cases (20.5%). Thirty-three scars (84.6%) were correctly characterized at the time they were identified without the need for further work-up. Six scars (15.4%) were considered to be more than expected for a typical post-biopsy scar and required further workup; four cases (10.3%) underwent short-term follow-up, and two cases (5.1%) required a biopsy for confirmation (Table 2). No malignancy was detected during available follow-up or additional evaluation (Figure 3).

**Figure 4:**
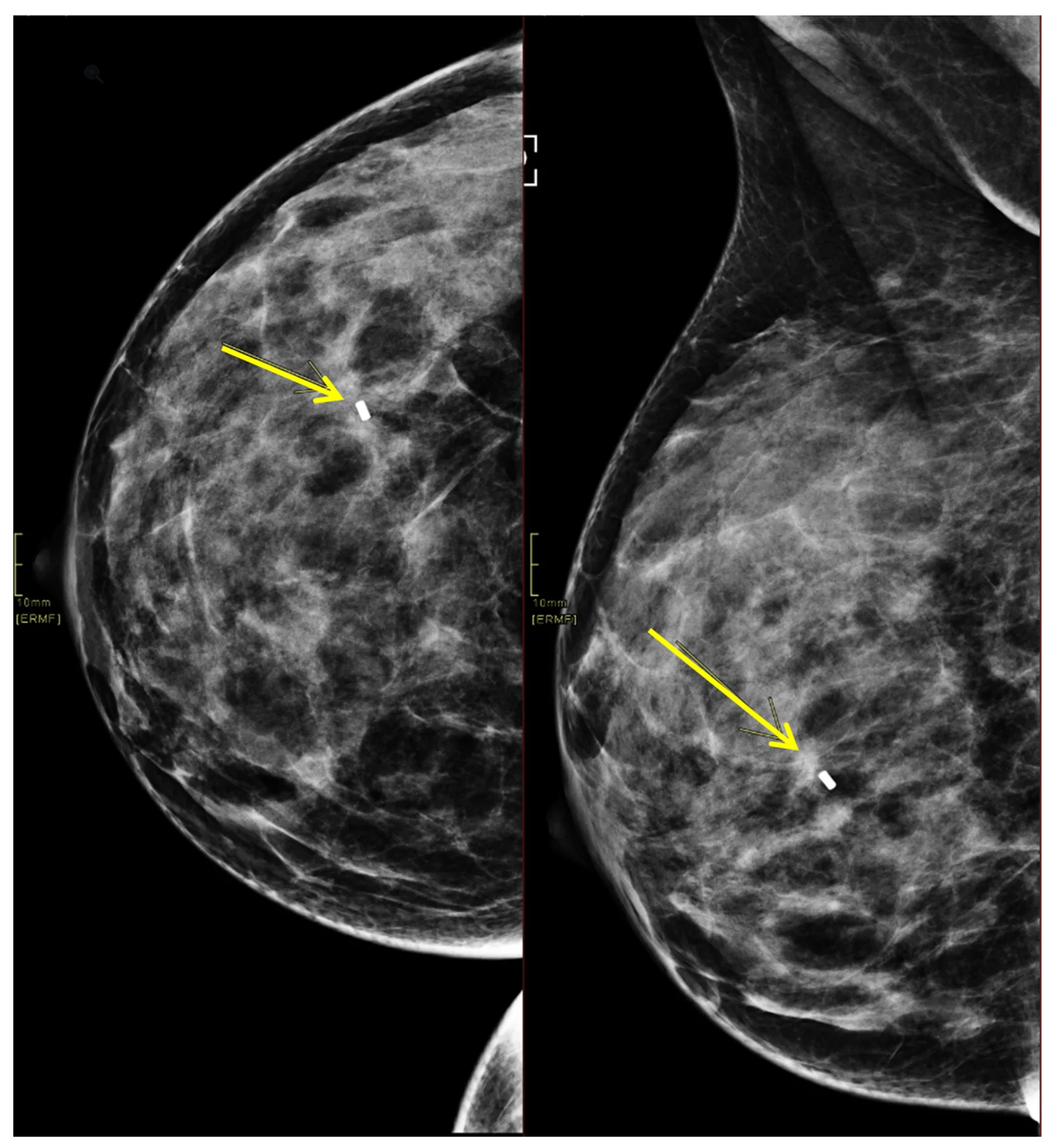
Right 2D CC and MLO of a patient showing scarring at the site of the biopsy and a clip marker noted in position. This same patient later underwent right mastectomy due to malignancy unrelated to the lesion biopsied.

Within our sample, five patients did not have a follow-up mammogram beyond the one where a scar was detected and, as such, were excluded from the Kaplan–Meier analysis of scar evolution. One of these patients developed cancer elsewhere in the breast and underwent mastectomy (Figure 4), three were entirely lost to follow-up, and one had a follow-up MRI done that showed no enhancement at the biopsy site.

At the last follow-up, the scar had regressed in 22 cases (56.4%) and was stable in 11 cases (28.2%). Only one case (2.6%) showed progression, for which an MRI was subsequently performed and showed no enhancement. The mean time to last follow-up was 59.11 ± 24.04 months, with a median duration of 60.0 months [IQR: 44.0–75.0] and a range from 12 to 108 months (Table 3).

**Figure 5:**
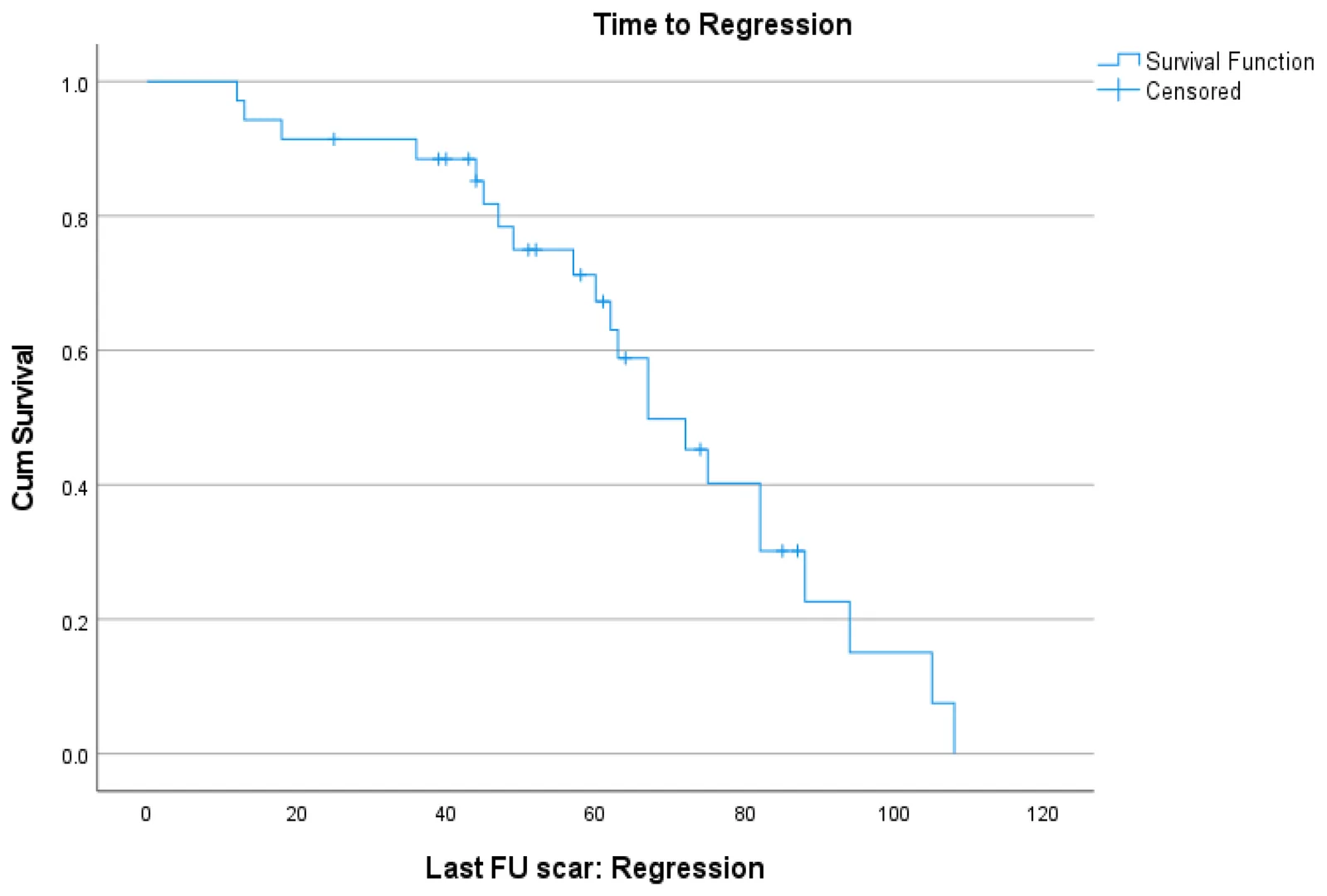
Kaplan–Meier curve showing time to scar regression in months after vacuum-assisted breast biopsy.

The Kaplan–Meier analysis was used to estimate time to scar regression among the 34 cases with available longitudinal follow-up. The time origin was the date of VABB, and scar regression was considered the event of interest. Scars that remained stable or progressed at the last available follow-up were censored at the time of their last imaging assessment. A total of 22 regression events were observed. The estimated mean time to scar regression was 69.47 months (95% CI: 59.65–79.28), while the estimated median time to scar regression was 67.00 months (95% CI: 54.50–79.50). These findings suggest that scar regression may occur gradually over long-term follow-up, with approximately half of scars regressing within nearly 5.6 years (Figure 5 and Table 4).

Patients with non-regressed scars had a mean age of 53.00 ± 13.36 years, compared with 51.23 ± 11.92 years among patients with regressed scars, with no significant difference between groups (*p* = 0.790). Similarly, lesion size was comparable between groups, with a mean size of 15.44 ± 18.67 mm in the non-regressed group and 16.47 ± 18.18 mm in the regressed group (*p* = 0.894). Scar regression was also not significantly associated with lesion laterality (*p* = 0.855) or breast density (*p* = 1.000).

Regarding mammographic findings, calcifications were more frequent among non-regressed scars than regressed scars (47.1% vs. 27.3%), but this difference did not reach statistical significance (*p* = 0.201). Architectural distortion and mammographic asymmetry were rare findings and not significantly associated with regression.

MRI findings were similarly not significantly associated with scar regression. MRI non-mass enhancement was more frequent among regressed scars compared with non-regressed scars (40.9% vs. 23.5%), but the difference was not statistically significant (*p* = 0.318). MRI focus was observed only among regressed scars (13.6% vs. 0.0%), but this difference was also not significant (*p* = 0.243).

## 4. Discussion

This study describes the imaging characteristics, longitudinal evolution, and clinical outcomes of documented post-biopsy scars following VABB in patients with benign pathology, contributing to the limited literature on the imaging characteristics and clinical implications of post-biopsy scarring. Only 15.4% of patients with documented post-biopsy scars had to undergo additional testing, including short-term follow-up or biopsy, and no malignancy was identified in association with the documented post-biopsy scars, consistent with previous findings that scarring is largely a benign and self-limiting process [13].

Our results align with prior reports by Yazici et al. [10] and Schaefer et al. [6], which noted scarring on follow-up mammograms in 4.3–16% of benign breast biopsies, typically presenting as vague asymmetries or architectural distortions at the biopsy site. These imaging appearances may overlap with those of malignancies that are identified as asymmetry or architectural distortion, with a positive predictive rate of 42.9% and 74.5%, respectively [14,15]. In our cohort, the majority of scars were more visible on the 3D oblique compared to other views. The higher visibility on 3D mammograms underscores the added value of tomosynthesis in detecting subtle architectural distortion. Importantly, with the increasing use of tomosynthesis in both the screening and diagnostic settings, it is crucial for radiologists to recognize normal post-biopsy changes that may be more frequently demonstrated by this technique to avoid misdiagnosing them as suspicious lesions.

One case illustrates the clinical implications of clip displacement. The patient initially underwent biopsy for suspicious calcifications, which were benign on histopathology. On follow-up imaging, a prominent and irregular post-biopsy scar was identified at the original biopsy site, while the clip had migrated away from this location. Despite the atypical scar appearance, a repeat biopsy of the associated distortion confirmed a benign process. This case, also demonstrating benign findings on MRI, highlights that in instances of clip displacement, the biopsy-related scar will not appear adjacent to the clip but instead at the true biopsy site (Figure 3). It reinforces the importance of recognizing that clip migration can occur and should be accurately documented in the initial post-biopsy mammogram, and that post-biopsy scarring should be assessed in relation to the original biopsy site rather than the final clip position. Notably, we found no significant difference in scar visibility based on whether the location was indexed to the biopsy site or the clip marker, supporting the utility of both as reliable landmarks provided that there was no clip displacement [6,16,17].

Scar visibility and evolution did not correlate significantly with lesion size, location, breast density, or imaging appearance of the initial lesion, echoing the findings of previous studies such as those of Lamm and Jackman [11] and Yazici et al. [10]. Moreover, no significant relationship was found between scar visibility and the delay in follow-up imaging, indicating that the scarring process is largely independent of follow-up timing within the 2–49 month window observed in our study.

Our study is one of the few that follows the progression of post-biopsy scars, where most cases regressed with a mean time of 69.47 months. The studies done by Lamm and Jackman and Yazici et al. did not extensively study this aspect, but were able to describe cases where they regressed or were stable [10,11]. This may help set an expectation on how long it may take for such scars to regress.

Our findings reinforce that while post-biopsy scars can mimic suspicious lesions on imaging and may occasionally warrant additional testing as short-term follow-up or tissue diagnosis, particularly in dense breasts, they are overwhelmingly benign and will, in around half of the cases, regress over time. Routine imaging remains adequate for accurate assessment, but radiologists must be attuned to the typical imaging appearance of post-biopsy scars to prevent unnecessary interventions.

The clinical relevance of biopsy-induced scarring extends beyond diagnostic accuracy. Scars may mimic or obscure underlying malignancies, particularly in high-risk patients or those undergoing surveillance imaging. Thus, comprehensive documentation of biopsy sites and correlation with clip marker location is essential for longitudinal interpretation.

This study is subject to several limitations. This first includes the modest sample size and retrospective design, where patients were only included if post-biopsy scarring was documented in follow-up imaging reports. The study was therefore affected by selection and reporting bias and cannot determine the true incidence of scar formation after VABB. The relatively small sample size and small subgroup counts require cautious interpretation of the statistical comparisons. The analyses were intended to be descriptive and exploratory, rather than to establish definitive predictors of scar formation or regression. In addition, patients with malignant or high-risk lesions requiring surgical excision were excluded to isolate cases of VABB-induced scarring. This approach focuses on benign post-procedural findings but limits the generalizability of results to benign cohorts. Moreover, histologic correlation of scar tissue was limited to the small subset of patients who underwent re-biopsy. Prospective studies with standardized imaging intervals and larger cohorts would help further elucidate the natural history of post-biopsy scarring and its diagnostic implications. It is also important to mention that, although all imaging studies where a scar was reported were reviewed by a single fellowship-trained breast radiologist, which ensured consistency, this design may introduce expectation bias and prevent assessment of inter-reader variability, and is acknowledged as a study limitation. Moreover, differences in VABB device technology, needle gauge, sampling technique, and biopsy protocol may influence post-biopsy changes, but these could not be evaluated in the present cohort where all patients had their biopsies performed using the same technology, needle gauge, and biopsy protocol.

Future research should build on these findings through larger, multicenter studies with prospective designs and standardized follow-up intervals to better characterize the evolution of post-biopsy scarring. Expanding patient diversity would improve generalizability, while exploring factors such as hormonal status, prior treatments, and breast density could clarify their impact on scar formation and visibility. Incorporating histologic correlation of scar tissue, where feasible, would also strengthen diagnostic conclusions. Such efforts would further refine our understanding of post-VABB changes and enhance interpretive accuracy in clinical practice. In conclusion, documented post-biopsy scars after VABB generally demonstrated benign behavior over time, with most remaining stable or regressing on follow-up imaging. While scars can mimic malignancies on imaging, awareness of their typical features and correlation with prior biopsy sites can help avoid unnecessary interventions. Ongoing surveillance and careful documentation remain essential for accurate interpretation and patient management.

## Figures and Tables

**Table 1:** Demographics, breast density, imaging modality, and pathology results of the sample studied.

Age at biopsy (in years)	Mean ± SD	51.97 ± 12.40	
	Median [IQR]	51.0 [41.5–61.0]	
	Min–Max	33–80	
Laterality	Right	19	48.7%
	Left	20	51.3%
Type of biopsy	Mammography-guided biopsy	16	41%
	MR-guided biopsy	23	59%
Class	BI-RADS 4	39	100%
Size (in mm)	Mean ± SD	16.03 ± 18.14	
	Median [IQR]	9.5 [6.0–19.3]	
	Min–Max	2–77	
Breast density	Heterogeneously dense	24	61.5%
	Extremely dense	6	15.3%
	Scattered fibroglandular densities	8	20.5%
	Predominantly fat	1	2.6%
Mammography finding	Calcifications	14	35.9%
	Asymmetry	1	2.6%
	Distortion	1	2.6%
MRI finding	Mass	7	17.9%
	Non-Mass	13	33.3%
	Focus	3	7.7%
Type of needle	9G	39	100%
Clip Marker	At biopsy site	38	97.4%
	Displaced	1	2.6%
Pathology result	Benign	39	100%

**Table 2:** Summarizes the findings of the first follow-up where post-biopsy scarring was detected.

Delay (months)	Mean ± SD	18.92 ± 12.88	
	Median [IQR]	14.5 [10.0–26.3]	
	Min–Max	2–49	
Mammogram view where scar was visible	2D CC	18	46.2%
	2D MLO	14	35.9%
	3D CC	8	20.5%
	3D MLO	32	82.1%
	US	17	43.6%
Scar requiring workup	No	33	84.6%
	Yes	6	15.4%
Scar requiring biopsy	No	37	94.9%
	Yes	2	5.1%

**Table 3:** Summarizes the findings of the last follow-up of the post-biopsy scar.

Status	Stable	11	28.2%
	Regressed	22	56.4%
	Progressed	1	2.6%
Last follow-up scar (months)	Mean ± SD	59.11 ± 24.04	
	Median [IQR]	60.0 [44.0–75.0]	
	Min–Max	12–108	

**Table 4:** Kaplan–Meier estimates of time to scar regression.

Outcome	Events, *n*	Censored, *n*	Mean Time, Months (95% CI)	Median Time, Months (95% CI)
Scar regression	22	12	69.47 (59.65–79.28)	67.00 (54.50–79.50)

## Data Availability

The data presented in this study are available on request from the corresponding author.

## Abbreviations

The following abbreviations are used in this manuscript:

CC	Craniocaudal
MLO	Mediolateral oblique
MRI	Magnetic resonance imaging
VABB	Vacuum-assisted breast biopsy
FU	Follow-up
